# Effects of fine-scale habitat quality on activity, dormancy, habitat use, and survival after reproduction in *Rana dybowskii* (Chordata, Amphibia)

**DOI:** 10.1186/s40850-022-00163-4

**Published:** 2023-01-16

**Authors:** Qing Tong, Wen-jing Dong, Xin-zhou Long, Zong-fu Hu, Zhi-wen Luo, Peng Guo, Li-yong Cui

**Affiliations:** 1 School of Biology and Agriculture, Jiamusi University, Jiamusi, 154007 China; 2 Northeast Agricultural University, Harbin, 150030 China; 3 Jiamusi Branch of Heilongjiang Academy of Forestry, Jiamusi, 154002 China

**Keywords:** Dormancy, Habitat use, Artificial refuges, Shade, Post-reproductive

## Abstract

Amphibians are facing population declines and extinctions, and protecting and supplementing refuges can help species survive. However, the microhabitat requirements of most species are unknown, and artificial shelters or burrows have not been well tested for amphibians. Some amphibians exhibit complex behaviour during the transition from post-reproductive dormancy to activity. However, little is known about the ecology, post-reproductive dormancy, and terrestrial activity of amphibians. Here, habitat site selection in experimental enclosures and the effects of shelters (stones, soil) and shade (with and without shade netting) on the activity, exposed body percentage, burrow depth, body-soil contact percentage, and survival of *Rana dybowskii* were investigated during post-reproductive dormancy and post-dormant activity. The results showed that *R. dybowskii* live individually under leaves, soil, stones or tree roots. Furthermore, although the dormant sites of frogs are significantly different, the distribution of male and female frogs in these sites is similar. Shading and shelter significantly affected the exposed body percentage, burrow depth and body-soil contact percentage of frogs compared with soil. In the stone group, soil and stone form the frog's refuge/burrow, whereas in the soil group, the refuge/burrow is composed entirely of soil. Even though the soil group has a deeper burrow and a larger area of soil contact with the body, it still has a higher exposure rate than the stone group. Frog activity frequency was affected by shelter and shade; the interaction of shelter and time and the interaction of shading and time were significant. The soil group had a higher activity frequency than the stone group, and the no-shade group had a higher activity frequency than the shade group. Shelter and shading differences do not significantly affect frog survival; however, the death rate during post-reproductive dormancy is lower than that during the active period.

**Supplementary Information:**

The online version contains supplementary material available at 10.1186/s40850-022-00163-4.

## Introduction

Amphibians are experiencing catastrophic population decreases and extinction due to infectious illnesses, habitat loss, climate change, invasive species, and chemical contaminants, underscoring the critical need for conservation [1]. Many amphibian species require a variety of habitat types to complete their life cycle [2]. Failing to conserve any of these habitats can lead to local extinction [2, 3]. The use of artificial refuges, captive breeding and reintroduction projects have played an important role in population management and species conservation [2]. Understanding the microhabitat preferences and minute activity patterns of animals within habitat patches is essential for both field management and predicting captive management settings in ex situ programs [4, 5].

There are many causes of amphibian declines, and in recent years, a growing body of research has focused on how amphibians are being affected by global climate change [5, 6]. In addition to raising average air and ocean temperatures, climate change has also increased the severity and frequency of extreme climatic occurrences [7]. Climate likely affects animal growth, development, foraging, hibernation, and breeding [7]. Permeable and exposed skin, unshelled eggs, complex life cycles, and ectothermic physiologies make amphibians more susceptible to aquatic and terrestrial temperature and precipitation variations [8]. It is crucial to lessen exposure to stressful situations to reduce vulnerability and the effects of climate change [5, 9, 10]. Since amphibians rely on external energy to control their body temperature, these refuges may help mitigate the consequences of climate change and extremes for amphibians [11, 12]. Previous research has demonstrated that manmade shelters or burrows are beneficial supplementary refuges for reptiles, but their use for amphibians has not been thoroughly examined [5, 13]. One difficulty is that habitat requirements for most species are virtually unknown.

Many amphibian species require a variety of habitat types to complete their life cycle [14]. Failing to conserve any of these habitats can lead to local extinction [2]. For the majority of endangered amphibians, elucidating their habitat needs is a crucial step in conservation strategies [15, 16]. The population trends and occurrences of amphibians are also heavily impacted by terrestrial habitat characteristics [13, 16]. However, the majority of amphibian population research relies on survey data collected from aquatic breeding habitats [13]. For example, pond-breeding amphibians (such as *Rana dybowskii*) require high-quality aquatic and terrestrial habitat [17]. After mating and spawning, both female and male *R. dybowskii* go from entering post-breeding dormancy to becoming active again sometime later, and *R. dybowskii* exhibits very complex behaviour, influenced by complex environmental factors [17]. Consequently, studying the migrations and habitat use of pond breeding amphibians can provide vital information on the quantity and type of terrestrial habitat needed, as well as crucial habitat features [3]. However, little research has been conducted on the ecology and behaviour of amphibians during post-reproductive dormancy and activity in terrestrial ecosystems; in particular, previous studies have not focused on habitat use during this period.

In place of natural refuges, artificial refuges are man-made buildings designed to provide secure areas for animals to use during breeding, dormancy, hibernation, or when seeking shelter [5, 13]. Around the world, artificial refuges are utilized to lessen the effects of numerous hazards to wildlife, such as habitat loss and degradation [5]. Natural refuges are generated by a variety of habitat structural components, such as tree bark, soil or stone crevices, and leaf shelters [12]. For wildlife, shelters are an important part of the animal environment [18]. Physical characteristics of the shelter (size, building materials) and surroundings (shade, vegetation, orientation of entrances, distance from resources) may influence animal predation or predator avoidance and survival [5]. The possibility that manmade refuges can successfully replace lost natural refuges can be increased by understanding the physical and environmental characteristics that a species prefers for its refuge [19]. Artificial shelters are useful extra refuges for amphibians, but their use for amphibians has not been well studied and the best practice for artificial refuge installation for wildlife conservation is still unknown [5, 6].

The brown frog (*R. dybowskii*) is mainly distributed in northeastern China, where it is widely hunted for its medicinal and commercial value and where the degradation of its habitat has drastically decreased its number [20]. *Rana dybowskii* has a significant fatality rate during the post-reproductive phase, which could be due to harsh weather in early spring and poor post-reproductive dormancy habitat conditions [21]. The high death rate of *R. dybowskii* during the post-reproductive period is a serious challenge to establishing captive breeding and reintroduction programs [22]. The survival of *R. dybowskii* exposed to harsh spring circumstances may depend on their ability to identify abundant climate refuges locally [11]. Can preserving or supplementing manmade and natural shelters, as well as building varied microclimate refuges, provide a buffer against life-threatening situations and suit the physiological needs of *R. dybowskii* that frequently inhabit these microhabitats [6, 11]?

In this study, we first studied post-reproductive dormancy site selection by *R. dybowskii* in experimental enclosures to determine its dormancy site preferences during post-reproductive dormancy; then, the effects of shelter and shade on the behaviour and survival of *R. dybowskii* following post-reproductive dormancy were investigated in a controlled experiment. Studying the optimal habitats of *R. dybowskii* during post-reproductive dormancy provides a reference for ecological research on amphibians.

## Materials and methods

### Experimental site and species

**Fig. 1:**
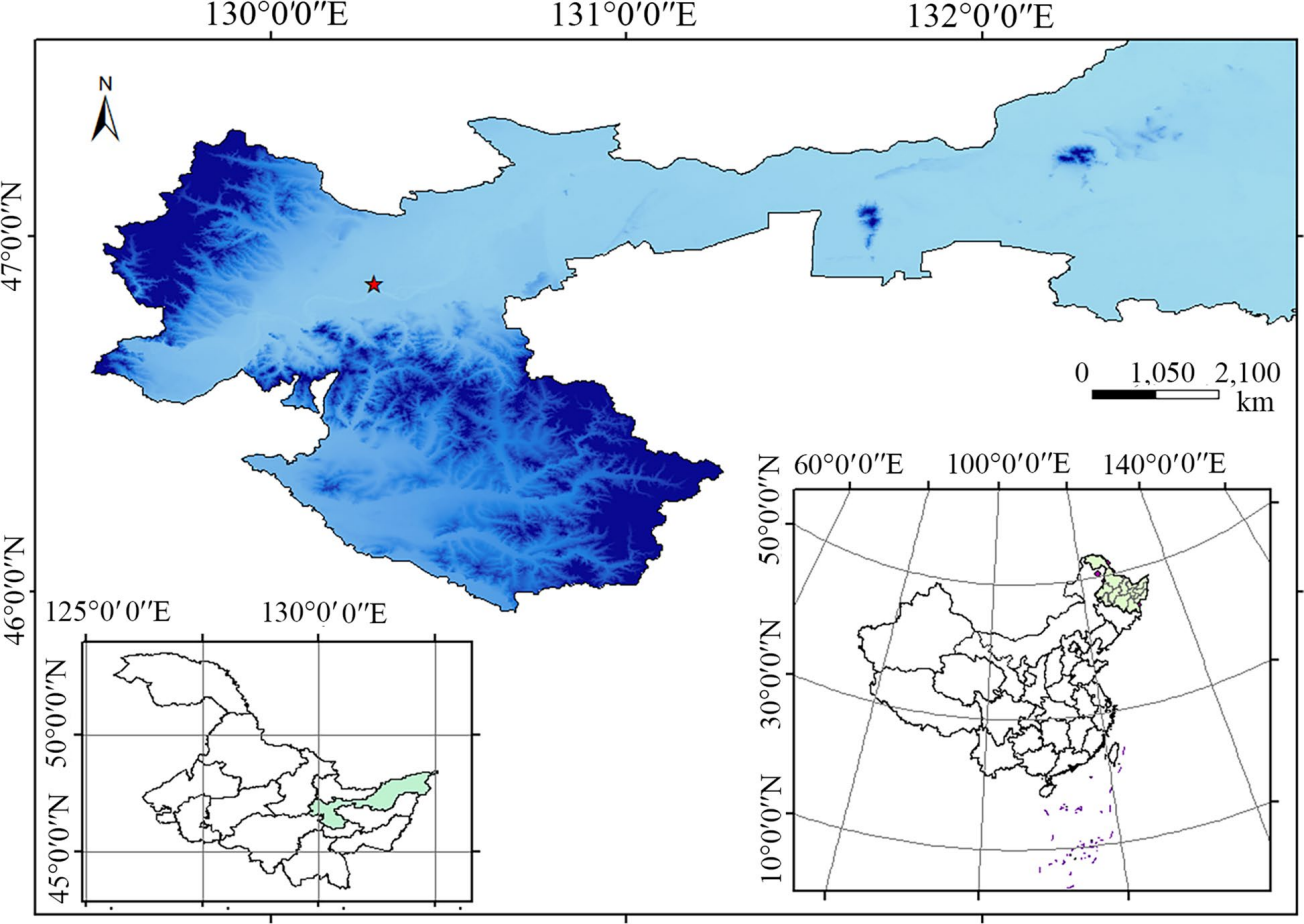
The experimental site was located in the suburbs of Jiamusi city. Jiamusi are situated near the northern foot of the Wanda Mountains and the western edge of the Three Rivers Plain. The best environment for a wildlife habitat is a mixed forest of trees, primarily consisting of poplar, birch, Quercus, linden, and willow, with a wide diversity of understory herbaceous plants

Experiments were conducted within the natural distribution zone of *R. dybowskii* in Jiamusi city, Heilongjiang Province, China (46°51′54″N, 130°17′32″E, altitude 80 m) (Fig. 1). Jiamusi has a moderate-temperature continental monsoon climate with long winters and short summers, a frost-free period of approximately 145 days, an annual precipitation volume of 510 mm, and an annual average temperature of 2.8 °C.

The brown frog used for the experiment came from a semimanufactured farm. The tadpole stage of the brown frog was raised by humans, while the young and adult frogs lived in the natural habitat of the forest frog. Each animal was tested once. At the end of the experiments, individuals were released at the capture site. The brown frogs had body masses of 19.31 ± 5.24 g (mean ± SD; males) and 25.11 ± 6.19 g (females) (in 2018; post-reproductive dormancy site selection) and 17.36 ± 4.57 g (males) and 29.65 ± 5.32 g (females) (in 2019; effects of shelter and shade on dormancy, behaviour and survival). All frogs were weighed before spawning. All experimental enclosures were located outdoors near spawning ponds and in the natural habitat.

### Methods

#### Post-reproductive dormancy site selection

##### Experimental design

To understand the specific dormant position of *R. dybowskii* in post-reproductive dormancy habitats, we chose 120 spawning frogs (male:female = 1:1) and placed them in the experimental enclosures to observe their dormant sites, activity and death.

##### Experimental enclosures and habitat manipulations

The enclosure was 900 m^2^ in zone and had spawning ponds. The depressions (spawning ponds) were spawning ponds for amphibian reproduction. At the beginning, the water at the centre of the spawning pond was 85.3 cm in depth and ~ 130 m^2^ in zone. The vegetation in the enclosure was well preserved and consisted of weeds and a small number of dwarf trees (59 trees), with approximately 340 m^2^ of the enclosure covered by fallen leaves. To prevent the frogs from escaping, we sealed the four site edges each with a plastic cloth fence (30.0 × 30.0 m^2^) supported by wooden sticks at an interval of 1–2 m. The bottom of each plastic cloth was embedded in soils, and the fence floor was approximately 1.0 m high.

##### Experimental procedure

Experiments were conducted in April and May 2018. On 10 April, vigorous wild frogs were placed in a spawning pond to lay eggs. After approximately 2 days, the frogs had finished laying eggs (Fig. S1). On 12 April, 60 male and 60 female frogs were randomly selected and placed in an experimental enclosure. During the experiment, frog activity and foraging were observed, and the integrity of the fence was checked on a daily basis. Any dead frog was immediately removed from the enclosure. Dormant sites were recorded on 25 April, 1 May and 6 May 2018. From 20 to 25 May, we searched for *R. dybowskii*, took the frogs out of the enclosure, and calculated their survival rates (survival number).

#### Effects of shelter and shade on dormancy, behaviour and survival

##### Experimental design

Four groups were established, and all tested in triplicate. Each replicate involved 50 frogs (male:female = 1:1; total 600 frogs), and the four dormancy microhabitats (experimental enclosures) are described below. The total zone of the four experimental enclosures was 48 m^2^.

##### Experimental enclosures and habitat manipulations

I Stone shelter without shading group: A dormancy zone (6.0 × 2.0 m^2^) surrounded by a fence (T-fences, 60 cm high) was divided into three dormancy subzones (each 2.0 × 2.0 m^2^). Each of the subzones was regarded as one replicate.
  T-fences were installed around the dormant subzones to prevent the animals from escaping. The floor of the dormancy zone was covered by a layer of cobblestones (diameter 10–20 cm). The cobblestones were placed in ridges with a gap of 20 cm between them and the fence. A transparent plastic cloth wider than the dormancy zone was installed in the upper part (1.6 m) of the zone to protect it from rainfall. To prevent strong wind and animal escape, we placed another fence (the outer fence) outside the dormancy zone (Fig. S2).
II Stone shelter with shading group: The same conditions as microhabitat I were adopted except that the top of the dormancy zone was covered with straw matting to reduce light penetration and exclude rainfall.
III Soil shelter without shading group: The same conditions as those in microhabitat I were employed except there was no stone, and a 20 cm thick soil layer was added to the surface of the dormant zone.
IV Soil shelter with shading group: The same conditions as microhabitat III were set, except that the dormancy zone was covered by a thin straw mat that reduced light penetration and prevented rainfall.

In all dormancy zones, the ground surfaces were wetted to maintain ~ 40% soil moisture before the frogs entered dormancy, but no wetting was performed during the dormancy period.

##### Experimental procedure

During the experiments, the frog population was periodically examined, and dead frogs were immediately removed. The survival rate (survival number) of *R. dybowskii* was investigated on both 6 May and 25 May 2019.

The frogs were randomly and periodically sampled to examine their resting status, exposed body percentage, burrow depth and body-soil contact percentage. The exposed body percentage was computed as the proportions of the abdomen and back that were exposed (the abdomen and back were counted as 45% and 55% of the body, respectively). The body-soil contact percentage was computed as the proportion of the body in contact with soil and was estimated in the same way as the exposed body percentage. Burrow depth was defined as the distance between the top of the highest surrounding soil and the abdomen of the frog.

The occurrence frequency (activity frequency) at which frogs appeared above ground was observed daily from 10 April to 25 May. “Appearance above ground” was defined as a frog exposing more than 75% of its back. If this condition was met, the corresponding frog was recorded as “appearing on the ground” (Fig. S4). We recorded the number of frogs appearing on the surface every day at approximately 14:00. Experiments were conducted between 10 April and 20 May 2019, and the above indices were measured on 25 April, 1 May and 6 May 2019. The survival rate (survival number) of *R. dybowskii* was investigated on 6 May and 20 May separately.

### Statistical analysis

All results are expressed as the mean ± standard deviation (x ± SD). Fisher's exact test was used to compare habitat distributions and survival rates in males and females. The Scheirer Ray Hare test (a nonparametric test) was used to analyse a two-way factorial design for the effect of shelters and shading on the body exposure rate, body-soil contact rate, and burrow depth, and the Nemenyi test was used for post hoc analysis.

Generalized estimating equations (GEE) were used to analyse the repeated-measures design for the effect of shelters, shading and time by selecting the frequency of activity at four time points: the beginning of post-reproductive dormancy (11 April), during post-reproductive dormancy (25 April and 10 May), and at the end of post-reproductive dormancy (25 May). The same method was used by selecting the surviving number at two time points: day 1 (6 May) and day 2 (20 May). Least-squares means estimated from GEE were used for pairwise post hoc multiple comparisons while applying the Bonferroni method to calculate the adjusted p values.

All statistical analyses were performed utilizing R software (version 3.5.3), the Scheirer Ray Hare test was conducted via the rcompanion package [23], and the generalized estimating equations (GEE) and post hoc tests were conducted with the gee pack and emmeans packages, respectively [24]. *P* < 0.05 indicated a significant difference.

## Results

### Post-reproductive dormancy site selection

During the experimental period, from 8 April to 30 May, the overall rise of the land at the experimental site was slow but slightly fluctuating (Fig. S4). *Rana dybowskii* tended to rest in dark and wet conditions at low temperatures (> 0 °C) in cool zones with small day-night temperature differences. The majority of the frogs entered dormancy alone, with all four limbs bowed. The ventral surfaces of the forelimbs were positioned behind the anterior borders of the eyes; the frogs' eyes were tightly closed, and they breathed slowly.

**Fig. 2:**
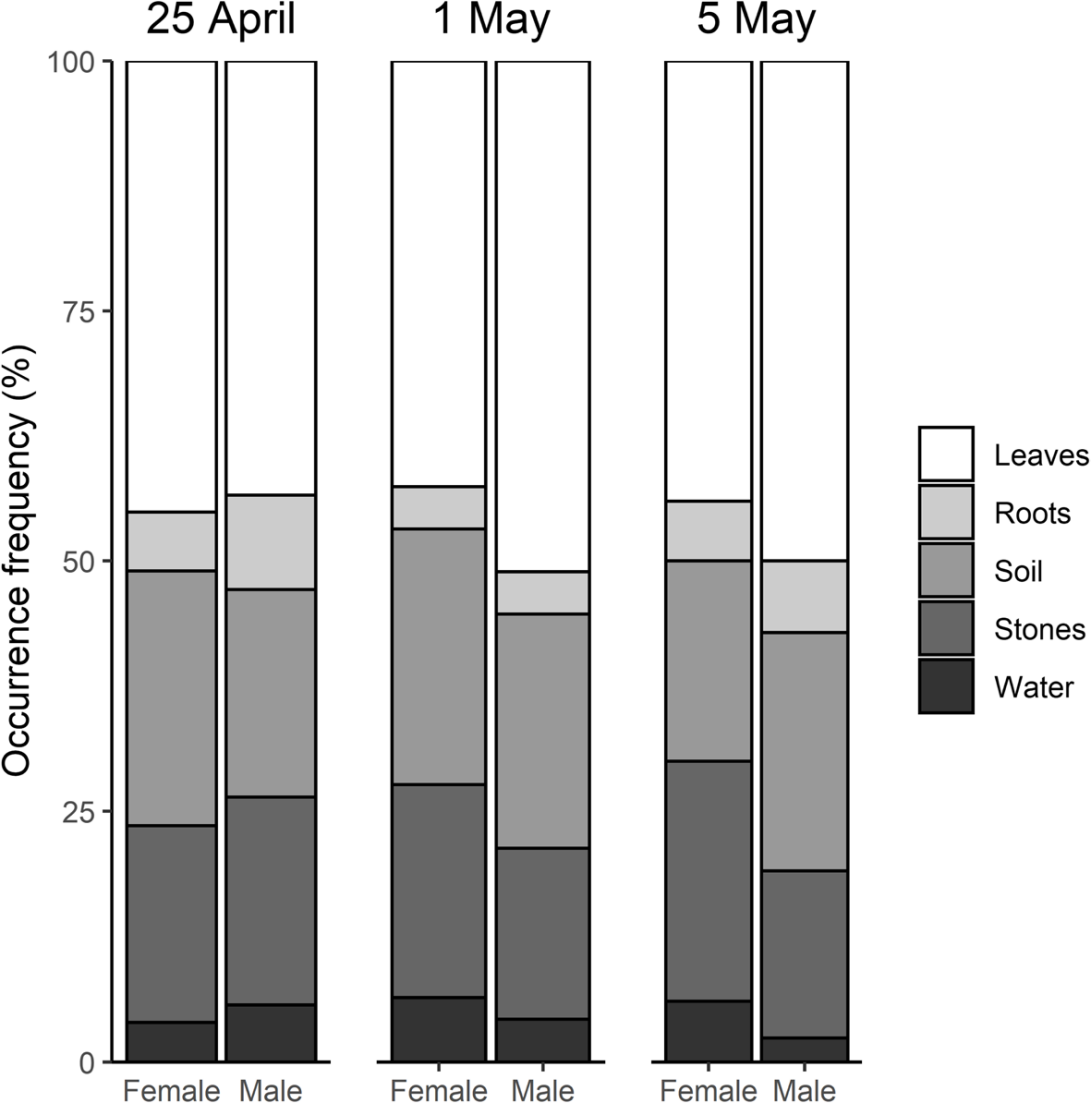
Site selection during post-reproductive dormancy in *R. dybowskii*. The majority of the frogs rested beneath leaves, beneath the soil, and beneath stones; nevertheless, a minor number rested beneath wate or beneath roots

There was a statistically significant difference in different frog dormant sites (*F* = 115.090, df = 4, *P* < 0.01). The majority of the frogs rested beneath leaves (female: 36.11%; male: 38.89%), beneath the soil (female: 19.44%; male: 17.78%), and beneath stones (female: 17.78%; male: 14.44%); nevertheless, a minor number rested beneath water (female: 4.44%; male: 3.33%) or beneath roots (female: 4.44%; male: 5.55%) (Fig. 2).

Male and female frogs did not differ in their habitat selection. The occurrence distribution of male and female frogs in these dormant sites did not differ significantly on 25 April, 1 May, or 5 May (Fisher’s exact test, *P* = 0.943, *P* = 0.937, and *P* = 0.824, respectively; Fig. 2).

There was no difference between male and female frogs in terms of survival rates. On 25 May, a total of 112 frogs were found, with 37 females and 39 males surviving and 18 females and 18 males dying. The difference between female and male survival (67.27% (37/55) vs. 68.42% (39/57)) was not significant (Fisher’s exact test, *P* = 1.000).

### Effects of shelter and shade on dormancy, behaviour and survival

The temperatures in the experimental enclosures all rose in a fluctuating manner from April to May (Fig. S4). The frogs instantly found hibernation locations, hiding in soil holes and under stones. They dug caverns and increased soil contact. During dormancy, animals dug burrows with their fore and rear limbs (Fig. S3). The frogs were able to easily create burrows in soft and loose soils and hide in the gaps between the stones and between the stones and the soil.

In the stone group, soil and stone form the frog's refuge/burrow, whereas in the soil group, the refuge/burrow is composed entirely of soil. The results of the Scheirer Ray Hare test for the two-factor design with shelter and shading factors showed a statistically significant difference in the interaction of shelter and shading for body exposure rate, body-soil contact rate, and burrow depth (Scheirer Ray Hare test, H = 4.7, df = 1, *P* = 0.029; H = 7.2, d f = 1, *P* = 0.007; H = 17.3, df = 1, *P* < 0.001, respectively) (Table S1).

**Fig. 3:**
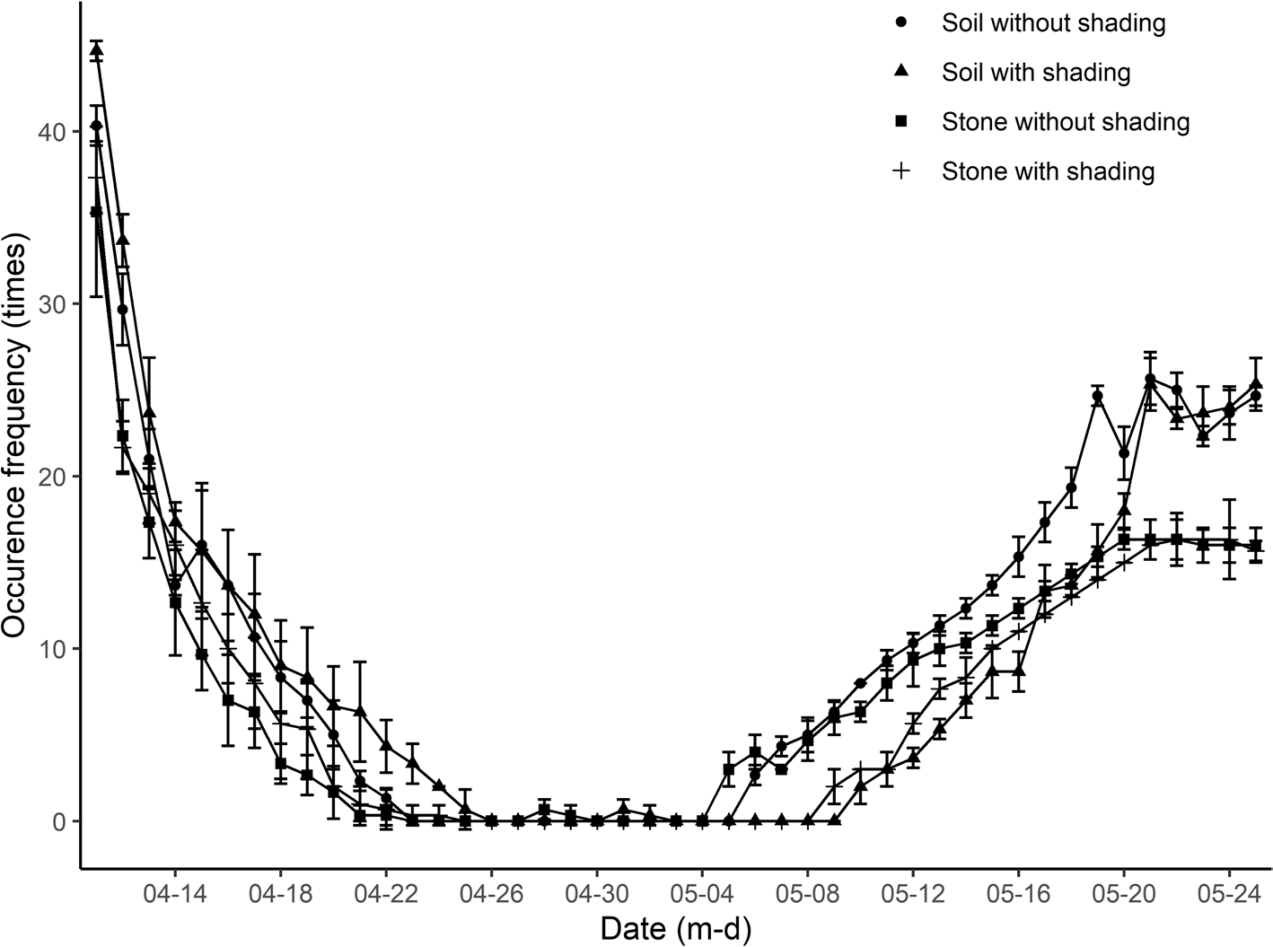
Occurrence frequency of post-reproductive dormancy of *R. dybowskii* under different shelters and with and without shade. In April, substrate and shade frogs were assigned to four habitats after spawning. Frogs found dormancy spots quickly. After many days, all frogs had found acceptable places to hide and fewer were on the ground. Frogs left dormancy and latency in early May. Over time, more frogs appeared

Post hoc tests showed that the body exposure percentages of the frogs were 23.26 ± 7.51% (soil without shading group), 30.21 ± 7.21% (soil with shading group), 7.35 ± 6.29% (stone without shading group), and 7.28 ± 6.76% (stone with shading group) (Fig. 5A). Comparing the body exposure percentage among all 4 groups, the only nonsignificant difference was between the soil without shading and soil with shading groups (Nemenyi test, χ^2^ = 0.017, df = 3, *P* = 0.999; Table S2, Fig. 3a).

The soil without shading group had a higher body-soil contact percentage than the stone without shading group. The body-soil contact percentages of the frogs were 73.54 ± 5.67% (soil without shading group), 67.26 ± 10.52% (soil with shading group), 61.50 ± 7.28% (stone without shading group), and 61.43 ± 3.98% (stone with shading group) (Fig. 5B). Comparing the body-soil contact percentage among all 4 groups, the only nonsignificant difference was between the soil without shading and soil with shading groups (Nemenyi test, χ^2^ = 0.889, df = 3, *P* = 0.828; Table S2, Fig. 3b).

The soil group had a deeper burrow depth than the stone group. The burrow depths of the frogs were 3.93 ± 0.35 cm (soil shelter without shading group), 3.42 ± 0.41 cm (soil shelter with shading group), 2.33 ± 0.28 cm (stone shelter without shading group), and 2.09 ± 0.35 cm (stone shelter with shading group) (Fig. 3c). There were significant differences among all four groups (Kruskal–Wallis rank sum test: χ^2^ = 186.524, df = 3, *P* < 0.01; Table S2, Fig. 3).

Even though the soil group had a deeper burrow and a larger area of soil contact with the body, the soil group still had a higher exposure rate than the stone group (Fig. 3). Comparing the body exposure percentage, body-soil contact percentage, and burrow depths among all 4 groups, the only nonsignificant difference was between the soil without shading and soil with shading groups (Nemenyi test, χ^2^ = 4.791, df = 3, *P* = 0.188; Table S2, Fig. 3c).

**Fig. 4:**
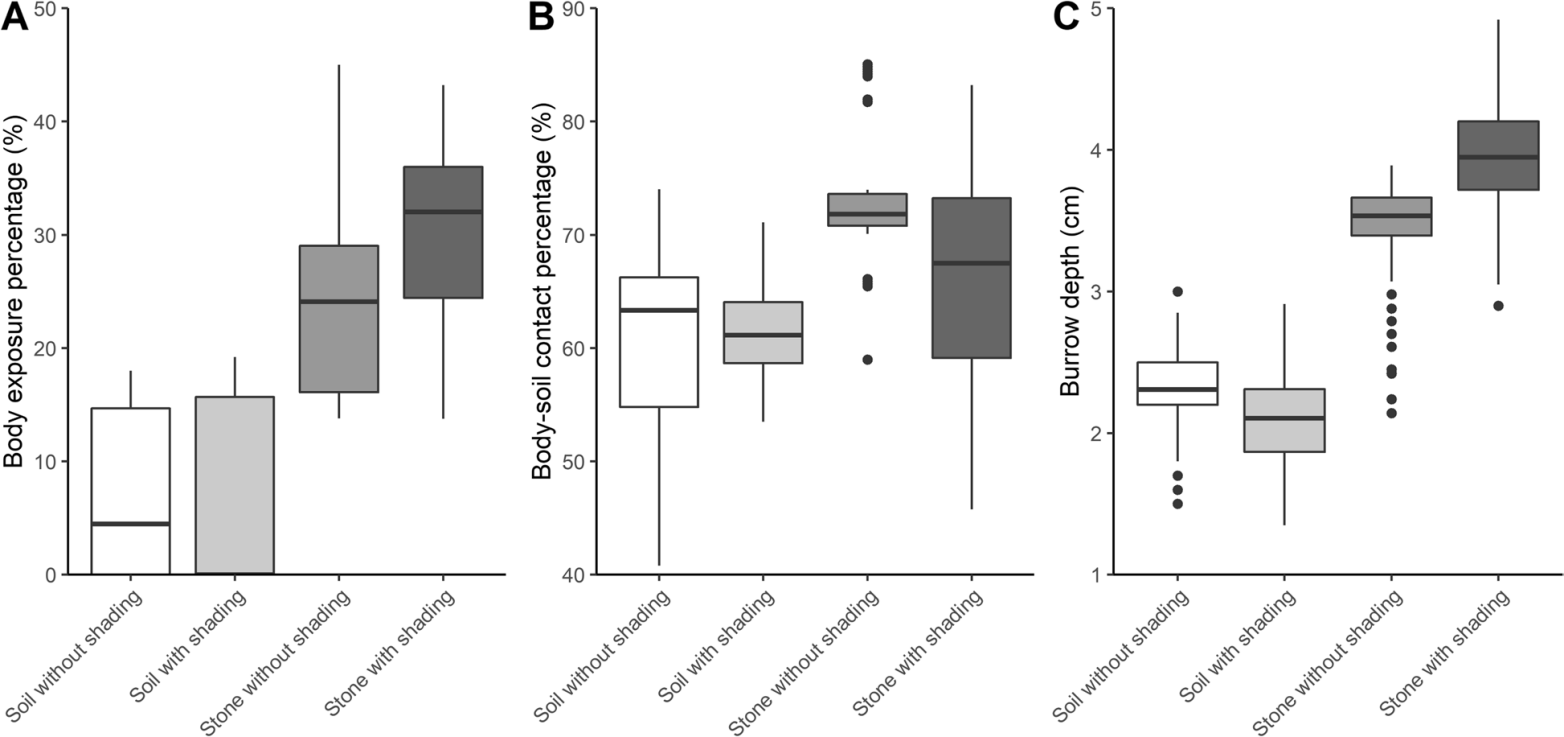
Effects of shelter and shade on the percentage of exposed body, body-soil contact percentage and depth of burrow of *R. dybowskii*. Ray Hare test for two-factor design with shelter and shading demonstrated a statistically significant interaction for body exposure rate, body-soil contact rate, and burrow depth

Figure 4 shows that one week later, practically all frogs had hidden and were rarely seen. On 5 May, the frogs moved (Fig. 4). GEE was used to investigate the influence of shelter, shading and time by selecting the frequency of activity at four time points (Table S3). The results indicated that the interaction of shelter and time and the interaction of shading and time were significant (*P* < 0.05; Table S4). Different times and shelters/shades resulted in different activity frequencies.

At some points in time (11 April and 25 May), frogs appeared more frequently in the shelter soil than in the shelter stone. In the comparison between shelter stone and soil, behaviour towards frogs was different at the beginning of post-reproductive dormancy (11 April) (Pairwise comparison of Least Square Means, adjusted *P* < 0.05), was not different during post-reproductive dormancy (25 April and 10 May) (Pairwise comparison of Least Square Means, *P* > 0.05) and was different at the end of post-reproductive dormancy (25 May) (Pairwise comparison of Least Square Means, adjusted *P* < 0.05) (Table S5).

At some points in time (11 April, 10 May, and 25 May), frogs appeared more frequently in the nonshade group than in the shade group. Comparisons between shade and nonshade on frog behaviour were different at the start of post-reproductive dormancy (11 April) (Least Square Means, adjusted *P* < 0.01); there was no difference during post-reproductive dormancy (25 April) (Pairwise comparison of Least Square Means, adjusted *P* > 0.05) and during post-reproductive dormancy (10 May) (Pairwise comparison of Least Square Means, adjusted *P* < 0.05); and at the end of post-reproductive dormancy (25 May), soil without shade was significantly different from soil with shade (Pairwise comparison of Least Square Means, adjusted *P* < 0.05), and stone without shade was not significantly different from stone with shade (Pairwise comparison of Least Square Means, adjusted *P* > 0.05) (Table S6).

**Fig. 5:**
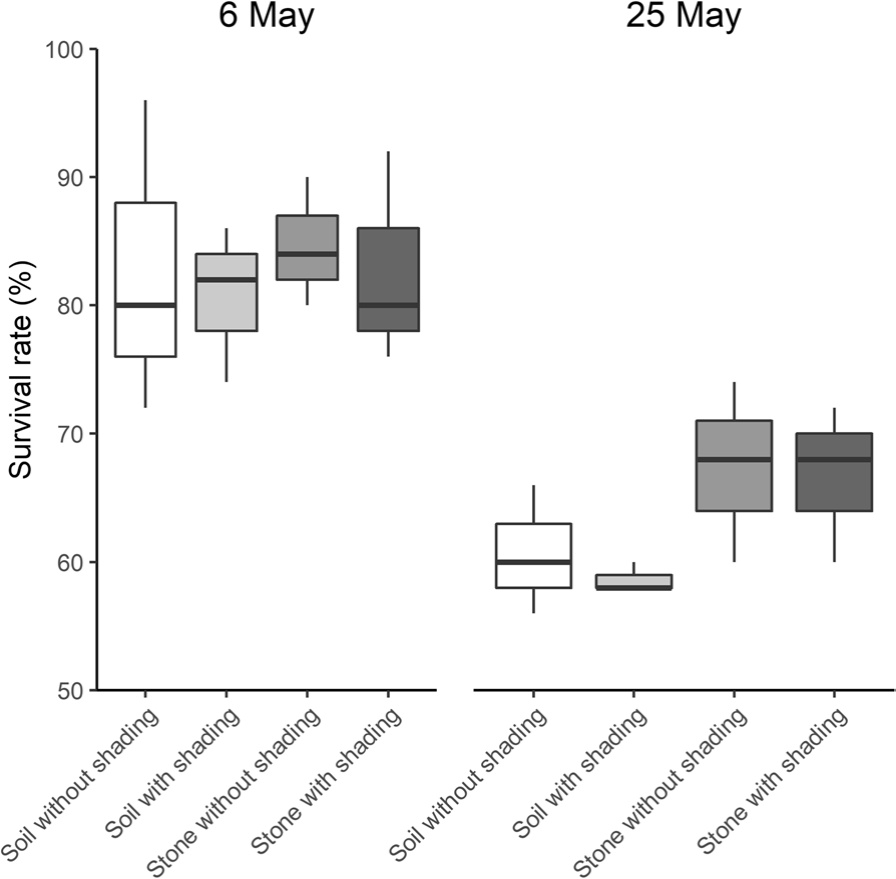
Effects of shelter and shade on the survival of *R. dybowskii*. The survival rates were measured at the end of most of the frogs' post-reproductive dormancy and after the end of the experiment. Data are displayed as the average ± SD

GEE was used to investigate the influence of shelter, shading and time on the survival rate of activity at two time points (Table S4). The survival rate of frogs did not differ among shelter/shade types (*P* > 0.05; Table S7). During dormancy (6 May, day 1), the survival rates were 82.67 ± 12.22% (soil group), 80.67 ± 6.11% (soil with shading group), 84.67 ± 5.03% (stone group), and 82.67 ± 8.32% (stone + shading group) (Fig. 5). By the end of the experiment (20 May, day 2), the survival rates were 60.67 ± 5.03% (soil without shading group), 58.67 ± 1.15% (soil with shading group), 67.33 ± 7.02% (stone without shading group) and 66.67 ± 6.11% (stone with shading group) (Fig. 5).

The survival rate of frogs was reduced in the post-dormancy activity period. The difference between day 1 (6 May) and day 2 (20 May) was statistically significant (pairwise comparison of least square means, adjusted *P* < 0.05), with the number of surviving frogs being smaller on day 2 (Table S8).

## Discussion

Amphibians are facing population declines and extinctions, and protecting and supplementing refuges can help species survive [22]. However, the microhabitat requirements of most species are unknown, and artificial refuges have not been well tested for amphibians [5, 22]. In this study, we first evaluated the post-reproductive dormancy site selection of *R. dybowskii* in experimental enclosures to determine its dormancy site preferences. We then investigated the effects of shelter and shade on *R. dybowskii* behaviour and survival following post-reproductive dormancy.

### Effects of temperature on habitat selection

Among the conditions that influence the activity or distribution of frogs, the most important may be temperature and water [25, 26]; in particular, low temperature may be a major inducer of post-reproductive dormancy in *R. dybowskii* [21]. In the present study, *R. dybowskii* live mainly under leaves, soil, and stones, and different dormant sites were significantly different. According to wood frog post-breeding habitat utilization, frogs prefer locations with deeper leaf litter layers, warmer air, and less humidity and light [27]. Post-breeding adult wood frogs rarely migrate long distances and seek areas near water to avoid desiccation and predators [16]. The habitat of post-breeding salamanders was positively associated with slash cover and negatively associated with grass, tree basal area, and humidity, and salamander habitats had leaves that were deeper and more humid [28]. Despite the similarity of the microhabitats chosen by these animals, there are differences. It is possible that other animals choose these habitats for feeding and living, whereas brown frogs select habitats for dormancy. In this study, in the early stage of post-reproductive dormancy, the average temperature over a 20-d period was lower than 10 °C (Fig. S3), while *R. dybowskii* chose a cool and hidden microhabitat for dormancy instead of an exposed habitat with a faster temperature rise [11, 29]. These places are not exposed to sunlight, the temperature is lower than that of unobstructed places, the temperature fluctuations are relatively small, and the humidity is greater.

Many species, including *R. dybowskii*, require both aquatic and terrestrial habitats, yet there is insufficient information on patterns of fine selection in these habitats [30]. Moisture is a necessary requirement for nearly all amphibians [26, 31], which select dormant habitats based on both humidity and temperature, especially humidity [22]. In the present study, after spawning, *R. dybowskii* was mostly dormant in terrestrial habitats near the spawning pools. Most *R. dybowskii* hibernate in water; young *R. dybowskii* that have not reproduced still hibernate in water [32]; however, in this study, *R. dybowskii* rarely chose to hibernate in water after breeding in water. In spring, variable water temperatures do not provide a cool resting environment for *R. dybowskii* [33], and *R. dybowskii* continuously enters and departs the water, which may not be conducive to dormancy. Away from water, amphibians are at risk of desiccation, and the nearby spawning pond provides many suitable humidity gradients, which may provide a suitable humidity environment after breeding; thus, sites that they choose to occupy are likely to contain elements that reduce this risk [34].

### The effect of temperature (shade) on frog behaviour

Temperature impacts animals, especially ectotherms, in all areas of their existence and survival [9]. Latitude, altitude, weather, and habitat composition affect organism temperatures [22, 35]. Our research has shown that there is no difference in the survival rate of frogs under different shade and time conditions. Some studies have shown that shade affects amphibian populations, and it may be that shade affects amphibians differently at different developmental levels. Captive-bred and reintroduced dusky gopher frogs prefer open canopies and rich ground plants and have a high survival rate [2]. Comparing the reactions of six species of tropical amphibian tadpoles inhabiting thermally opposed open and shade habitats revealed that open habitat species frequently adapt more rapidly than shade habitat species [9]. Tree removal from historically open-canopy ponds promotes the recovery of open-habitat species [6]. In contrast, shade can impact the growth rate of gopher frogs, prolong the larval phase, lower survival rates, and diminish the quality of metamorphosis [36]. The amount of heat-related mortality at some propagation locations can be decreased by increasing the canopy cover [6]. There are differing views on the effects of shade on amphibian behaviour and populations, especially as climate change is causing an accelerated increase in average temperatures and extreme heat events [9].

During dormancy (between 25 April and 10 May), there was no significant effect of shading on the activity. Shade has no discernible impact on *R. dybowskii* activity at this time since it has previously entered dormancy to find a suitable inactive spot and goes dormant [22]. *Rana dybowskii* may be mainly affected by temperature (they hibernate mainly in shelters); low temperature inhibits frog activity [25]. Our studies have shown that frogs have different activity frequencies under different shade and time conditions, with a particularly pronounced performance after 4 May. Captive-bred and reintroduced dusky gopher frogs prefer open canopies and rich ground plants [2]. Terrestrial ectotherms adjust their body temperatures behaviourally to maintain ideal body temperatures by varying their daily or seasonal use of shady or sunny microhabitats [37, 38]. To maintain optimal body temperatures, *R. dybowskii* behaviourally regulates body temperature by altering the daily or seasonal use of shaded or sunny microhabitats [37, 39]

### Refuges and burrow

Amphibians use refuges or shelters for a variety of reasons, including ambush feeding, buffering, reducing osmoregulatory and thermal stresses, and predator avoidance [40]. Often to lessen the threat of predation or to improve adaptability and access to resources, animals must consider the physical characteristics of the shelter (such as size and construction materials), as well as the surrounding environmental conditions (vegetation, entrance orientation, and distance to resources) [5]. In the present study, the role of refuges may be different during dormant and active periods.

Almost all *R. dybowskii* in our study used shelters (rocks and loose soil holes) during the dormant period; some frogs even dug holes to make shelters during dormancy (Fig. 4). The typical harsh spring conditions of rain, wind and cold do not provide favourable conditions for *R. dybowskii* to end their dormancy (Green et al. 2016). Thus, the fate of *R. dybowskii* exposed to harsh springtime conditions will depend on whether they can find locally abundant refuges that buffer against life-threatening conditions [11, 12]. Although we have not examined the microclimate within the shelter, previous research indicates that tree hollows create a more stable microclimate than their surroundings, sustaining lower temperatures and greater humidity during the day and higher temperatures and lower humidity at night [41]. Previous research has demonstrated that common frog microhabitats (e.g., soil, tree holes) can reduce exposure to temperature extremes by 14–31 times, hence reducing population vulnerability by a factor of 108 and potentially reducing death during extreme weather events [11]. By providing a proper microclimate, refuges or shelters may have thermoregulatory and osmoregulatory effects; they may also minimize physiological stress in terrestrial anurans induced by heat, cold, and drought [42–44]. During the early stages of dormancy, the shelter may also function as concealment in areas where *R. dybowskii* repose, allowing them to evade predators and providing a safe environment for the frogs during dormancy [40].

In this study, the majority of frogs found refuge or shelter at the experimental site. Frogs in soil shelters were more active than those in stone shelters, but there was no difference in their survival rates. Previous research has demonstrated that individuals without shelter spend more time digging and that all frogs spend a considerable amount of time beneath shelter [40]. Significant impacts of shelter (plant provision) on captive red-eyed tree frog body size and growth rates suggest a fitness benefit [18]. Frogs hiding in underground shelters had a 22% higher survival rate than frogs not hiding underground compared to frogs released into burrows; frogs released at ponds had a 33% lower survival rate, spent less time underground, and moved farther and more often in search of shelter [2]. Several studies have found increased movement of amphibians on ground shelters without litter or on bare ground [45]. The soil shelters in this study were more likely to be damaged with wind and rain than the stone shelters, and the frog's cover was damaged and the frequency of activity increased. Our results are consistent with these studies, suggesting that bare ground and limited shelter availability may be important drivers of increased movement and decreased settlement in poor habitats, possibly in response to higher predation and desiccation risks [4].

The two strategies used by frogs to dig burrows to avoid desiccation appear to be different, and the type of soil has an effect on both strategies [8, 46]. In sandy or crumbly soils, anurans can easily burrow, and when the earth is dry, these frogs tunnel even deeper to remain below the dry front [46]. To prevent dehydration, frogs prefer to stay in moist soil, and several species can often dive to a depth of 90 cm [8]. Anurans that inhabit dense, clay-rich soils use a different technique. Anurans tunnel 10–30 cm below the soil's surface in this soil [47]. As the earth around them dries, the frogs construct a cocoon from exfoliated skin and mucus [46]. In this study, the soil cut-off was deeper than the rock-medium cave. It may be that loose soil is easier to burrow into. Amphibians may rehydrate by absorbing water from the soil around them; therefore, it was thought that when burrowing anurans were exposed to dry conditions, they would dig deeper into the shelter to find moisture and rehydrate [8, 46]. However, irregularly wet rocks tend to aid in water conservation, resulting in shallower borrows.

### Conservation implications

There are currently few meaningful management actions that will have a tangible effect on amphibians' vulnerability to climate change [6]. Numerous potentially beneficial but untested actions could be implemented into local or regional amphibian management plans, programs, and activities [6]. Examples include retaining or supplementing artificial and natural shelters to mitigate desiccation and thermal stress and adjusting the shade fabric covering habitats to maintain an ambient temperature [6]. The complexity of the environment, specifically shelter or the provision of cover, may be a crucial factor to consider when constructing enclosures for captive amphibian rearing [18]. Captive red-eyed tree frogs exhibit a satisfactory growth rate under covered conditions, indicating that providing shelters is preferable for this type of frog [18, 48]. For *Xenopus*, shelter cover appears to confer behavioural benefits but has no impact on the growth or body conditions of this type of frog, whereas for nonmodel taxa, shelter cover might exert positive effects on growth as well as behaviour [18, 40].

In this study, stones were used as a shelter during the dormant period for *R. dybowskii* in terrestrial habitats. It is necessary to consider a type of shelter conducive to a particular frog's dormancy. Previous research suggested that PVC pipes and cover boards could provide suitable shelter for some amphibians [6, 13]. The temperature change in PVC pipes is usually larger than that under natural shelters [6]. We recommend the use of a thin cystosepiment as a shelter or refuge, as cystosepiment has many advantages, such as it does not press down on *R. dybowskii*, does not irritate their skin, is easy to clean and disinfect, and is slow to conduct heat in direct sunlight. However, in the dormant state, many *R. dybowskii* individuals are crowded together, pressing against each other; if some individuals move, the remainder may be easily disturbed [49]. Therefore, more research is needed to better understand frog microhabitat needs and to evaluate different shelter designs [6].

## Conclusions

The results showed that *R. dybowskii* lives individually under leaves, soil, stones or tree roots. Furthermore, although the dormant sites of frogs were significantly different, the occurrence distributions of male and female frogs at these sites were similar. Shading and shelter significantly affected the exposed body percentage, burrow depth and body-soil contact percentage of frogs compared with soil. Frog activity frequency was affected by shelter and shade, and there are interaction effects between shelter + shade and time. Shelter and shading differences did not significantly affect frog survival; however, the death rate during post-reproductive dormancy was lower than that during the active period. These findings advance our understanding of amphibian behaviour ecology by demonstrating how the interaction of external factors and individual behaviour results in observed patterns of movement and habitat use.

## Supplementary Information

**Additional file 1: Figure S1.** Phenological time of *R. dybowskii* emerging from hibernation, post-reproduction dormancy, activity, and feeding. **Figure S2.** Diagrams of microhabitat II described in Section 2.2.2. **Figure S3.** The occurrence frequency of frogs was measured by their presence on the ground. **Figure S4.** The temperatures in the experimental enclosures. 

**Additional file 2: Table S1.** Scheirer Ray Hare test for two-factor design with shelter and shading demonstrated a statistically significant interaction for body exposure rate, body-soil contact rate, and ground hole depth. **Table S2.** Comparing the body exposure percentage, body-soil contact percentage and borrows depth among all 4 groups by Nemenyi test. **Table S3.** Used GEE to investigate the influence of shelter, shading and time by selecting the frequency of activity at four time points. **Table S4.** The results indicated that interaction of shelter and time, interaction of shading and time had significant. **Table S5.** Comparing frog behavioural differences in refuge stones and soil at four time points. **Table S6.** Comparisons between shade and non-shade on frog behaviour at four time points. **Table S7.** Used GEE to investigate the influence of shelter, shading and time by survival rate of activity at two time points. **Table S8.** The survival rate of frog was reduced in the post-dormancy activity period.

## Data Availability

All data generated or analysed during this study are included in this published article and its supplementary information files.

## References

[1] Tong Q, Cui LY, Bie J, Han XY, Hu ZF, Wang HB, Zhang JT (2021). Changes in the gut microbiota diversity of brown frogs (*Rana dybowskii*) after an antibiotic bath. BMC Vet Res.

[2] Roznik EA, Reichling SB (2021). Survival, movements and habitat use of captive-bred and reintroduced dusky gopher frogs. Anim Conserv.

[3] Brown C, Nowakowski AJ, Keung NC, Lawler SP, Todd BD: Untangling multi-scale habitat relationships of an endangered frog in streams to inform reintroduction programs. Ecosphere. 2021;12(10):e03799.

[4] Osbourn MS, Connette GM, Semlitsch RD (2014). Effects of fine-scale forest habitat quality on movement and settling decisions in juvenile pond-breeding salamanders. Ecol App.

[5] Cowan MA, Callan MN, Watson MJ, Watson DM, Doherty TS, Michael DR, Dunlop JA, Turner JM, Moore HA, Watchorn DJ (2021). Artificial refuges for wildlife conservation: what is the state of the science?. Biol Rev.

[6] Shoo LP, Olson DH, McMenamin SK, Murray KA, Van Sluys M, Donnelly MA, Stratford D, Terhivuo J, Merino-Viteri A, Herbert SM, Bishop PJ (2011). Engineering a future for amphibians under climate change. J Appl Ecol..

[7] Ruthsatz K, Dausmann KH, Peck MA, Glos J (2022). Thermal tolerance and acclimation capacity in the European common frog (*Rana temporaria*) change throughout ontogeny. J Exp Zool A Ecol Integr Physiol.

[8] Szekely D, Cogalniceanu D, Szekely P, Denoel M (2018). Dryness affects burrowing depth in a semi-fossorial amphibian. J Arid Environ.

[9] Turriago JL, Tejedo M, Hoyos JM, Bernal MH (2022). The effect of thermal microenvironment in upper thermal tolerance plasticity in tropical tadpoles. Implications for vulnerability to climate warming. J Exp Zool A Ecol Integr Physiol.

[10] Wei S, Sun S, Dou H, An F, Gao H, Guo C, Hua Y (2022). Influence of Pleistocene climate fluctuations on the demographic history and distribution of the critically endangered Chinese pangolin (*Manis pentadactyla*). BMC Zool..

[11] Scheffers BR, Edwards DP, Diesmos A, Williams SE, Evans TA (2014). Microhabitats reduce animal's exposure to climate extremes. Glob Change Biol.

[12] Romero GQ, Gonçalves-Souza T, Roslin T, Marquis RJ, Marino NA, Novotny V, Cornelissen T, Orivel J, Sui S, Aires G (2022). Climate variability and aridity modulate the role of leaf shelters for arthropods: a global experiment. Glob Change Biol.

[13] Michael DR, Blanchard W, Scheele B, Lindenmayer DB (2019). Comparative use of active searches and artificial refuges to detect amphibians in terrestrial environments. Austral Ecol.

[14] Valdez JW, Gould J, & Garnham JI. Global assessment of artificial habitat use by amphibian species. Biological Conservation. 2021;257:109129

[15] Huang Y, Zhao W, Ding L, Bao X, Wang J, Lin Y, Ran J, Yang D, Zou H, Liu J (2019). Habitat selection and genetic structure of the endangered frog species Odorrana wuchuanensis (Anura: Ranidae). Zoolog Sci.

[16] Taylor MED, Paszkowski CA (2018). Postbreeding movement patterns and habitat use of adult Wood Frogs (*Lithobates sylvaticus*) at urban wetlands. Can J Zool.

[17] Tong Q, Cui LY, Hu ZF, Du XP, Abid HM, Wang HB (2020). Environmental and host factors shaping the gut microbiota diversity of brown frog *Rana dybowskii*. Sci Total Environ.

[18] Michaels CJ, Antwis RE, Preziosi RF (2014). Impact of plant cover on fitness and behavioural traits of captive red-eyed tree frogs (*Agalychnis callidryas*). PLoS ONE.

[19] Reside AE, Briscoe NJ, Dickman CR, Greenville AC, Hradsky BA, Kark S, Kearney MR, Kutt AS, Nimmo DG, Pavey CR (2019). Persistence through tough times: fixed and shifting refuges in threatened species conservation. Biodivers Conserv.

[20] Tong Q, Liu X-N, Hu Z-F, Ding J-F, Bie J, Wang H-B, Zhang J-T (1912). Effects of Captivity and Season on the Gut Microbiota of the Brown Frog (*Rana dybowskii*). Front Microbiol.

[21] Tong Q, Cui L-Y, Liang C-B, Chuang W (2015). Effects of breeding pens on reproductive dormancy of Rana dybowskii. Heilongjiang Anim Husbandry Vet.

[22] Hammond TT, Curtis MJ, Jacobs LE, Gaffney PM, Clancy MM, Swaisgood RR, Shier DM (2021). Overwinter behavior, movement, and survival in a recently reintroduced, endangered amphibian Rana muscosa. J Nature Conserv.

[23] Neury-Ormanni J, Doose C, Majdi N, Vedrenne J, Traunspurger W, Morin S (2020). Selective grazing behaviour of chironomids on microalgae under. Sci Total Environ.

[24] Regterschot GRH, Bussmann JBJ, Fanchamps MHJ, Meskers CGM, Ribbers GM, Selles RW (2021). Objectively measured arm use in daily life improves during the first 6months poststroke: a longitudinal observational cohort study. J Neuroeng Rehabil.

[25] Vidal MA, Barría-Oyarzo I, Contreras C, Bacigalupe LDJ (2020). Geography, temperature, and water: Interaction effects in a small native amphibian. Physiol Biochem Zool.

[26] Pilliod DS, McCaffery RM, Arkle RS, Scherer RD, Cupples JB, Eby LA, Hossack BR, Lingo H, Lohr KN, Maxell BA (2022). Importance of local weather and environmental gradients on demography of a broadly distributed temperate frog. Ecol Ind.

[27] Rittenhouse TAG, Semlitsch RD (2007). Postbreeding habitat use of wood frogs in a Missouri oak-hickory forest. J Herpetol.

[28] Ryan KJ, Calhoun AJK (2014). Postbreeding Habitat Use of the Rare, Pure-Diploid Blue-spotted Salamander (Ambystoma laterale). J Herpetol.

[29] Hinderer RK, Litt AR, McCaffery M (2021). Habitat selection by a threatened desert amphibian. Ecol Evol.

[30] Baldwin RF, Calhoun AJK, deMaynadier PG (2006). Conservation planning for amphibian species with complex habitat requirements: a case study using movements and habitat selection of the wood frog rana sylvatica. J Herpetol.

[31] Walls SC, Barichivich WJ, Brown ME (2013). Drought, deluge and declines: the impact of precipitation extremes on amphibians in a changing climate. Biology.

[32] Tong Q, Cui L-Y, Hu Z-F, Du X-P, Abid HM, Wang H-B (2020). Environmental and host factors shaping the gut microbiota diversity of brown frog *Rana dybowskii*. Sci Total Environ.

[33] Tattersall GJ, Ultsch GRJ (2008). Physiological ecology of aquatic overwintering in ranid frogs. Biol Rev.

[34] Evans MJ, Scheele BC, Westgate MJ, Yebra M, Newport JS, Manning AD (2020). Beyond the pond: terrestrial habitat use by frogs in a changing climate. Biol Cons.

[35] Ortega Z, Mencia A, Perez-Mellado V (2017). Wind constraints on the thermoregulation of high mountain lizards. Int J Biometeorol.

[36] Burrow AK, Maerz JC (2021). Experimental confirmation of effects of leaf litter type and light on tadpole performance for two priority amphibians. Ecosphere.

[37] Ryan MJ, Latella IM, Giermakowski JT, Snell H, Poe S, Pangle RE, Gehres N, Pockman WT, McDowell NG (2016). Too dry for lizards: short-term rainfall influence on lizard microhabitat use in an experimental rainfall manipulation within a piñon-juniper. Functional Ecol.

[38] Rossi GS, Cramp RL, Wright PA, Franklin CE (2020). Frogs seek hypoxic microhabitats that accentuate metabolic depression during dormancy. J Experimen Biol.

[39] Heard G, Robertson P, Scroggie M (2008). Microhabitat preferences of the endangered Growling Grass Frog *Litoria raniformi*s in southern Victoria. Australian Zoologist.

[40] Walsh PT, Downie JR (2005). The effects of shelter availability and substrate quality on behaviour and post-metamorphic growth in three species of anurans: implications for captive breeding. Herpetol J.

[41] O'Connell C, Keppel G (2016). Deep tree hollows: important refuges from extreme temperatures. Wildl Biol.

[42] Tracy CR, Laurence N, Christian KA (2011). Condensation onto the skin as a means for water gain by tree frogs in tropical Australia. Am Nat.

[43] Heemeyer JL, Williams PJ, Lannoo MJ (2012). Obligate crayfish burrow use and core habitat requirements of Crawfish Frogs. J Wildl Manag.

[44] Seebacher F, Alford RA. Shelter microhabitats determine body temperature and dehydration rates of a terrestrial amphibian (*Bufo marinus*). J Herpetol. 2002;36(1);69–75.

[45] Manenti R, Melotto A, Denoël M, Ficetola GF (2016). Amphibians breeding in refuge habitats have larvae with stronger antipredator responses. Anim Behav.

[46] Booth DT (2006). Effect of soil type on burrowing behavior and cocoon formation in the green-striped burrowing frog, Cyclorana alboguttata. Can J Zool.

[47] Tracy CR, Reynolds SJ, McArthur L, Tracy CR, Christian KA (2007). Ecology of aestivation in a cocoon-forming frog, Cyclorana australis (Hylidae). Copeia.

[48] Rittenhouse TAG, Semlitsch RD (2009). Behavioral response of migrating wood frogs to experimental timber harvest surrounding wetlands. Can J Zool.

[49] Cui YY, Cui Y, Hou XD, Hu CG (2007). Study on reproductive dormancy technique of cultured *Rana dybowskii*. Pract For Technol.

