# Supplementary material for: Effects of fine-scale habitat quality on activity, dormancy, habitat use, and survival after reproduction in Rana dybowskii (Chordata, Amphibia)
Source: BMC Zool. 2023 Jan 16;8:1. doi: 10.1186/s40850-022-00163-4 (PMC10127375; doi:10.1186/s40850-022-00163-4)
Supplement: Supplementary file 1 — Additional file 1: Figure S1. Phenological time of R. dybowskii emerging from hibernation, post-reproduction dormancy, activity, and feeding. Figure S2. Diagrams of microhabitat II described in Section 2.2.2. Figure S3. The occurrence frequency of frogs was measured by their presence on the ground. Figure S4. The temperatures in the experimental enclosures. [file 40850_2022_163_MOESM1_ESM.docx]

**Supplementary material**

**Effects of fine-scale habitat quality on activity, dormancy, habitat use, and survival after reproduction in *Rana dybowskii* (Chordata, Amphibia)**

Qing Tong^1,3^, Wen-jing Dong^1^, Xin-zhou Long^1^, Zong-fu Hu^3^, Zhi-wen Luo^1^, Peng Guo^1^, Li-yong Cui^2*^

1. School of Biology and Agriculture, Jiamusi University, Jiamusi, 154007, China

2. Jiamusi Branch of Heilongjiang Academy of Forestry, Jiamusi 154002, China

3. Northeast Agricultural University, Harbin, 150030, China

* Correspondence:

Tong Qing

lxix@126.com

Li-yong Cui

cuiliy@163.cm

* Corresponding author. Mailing address: Jiamusi Branch of Heilongjiang Academy of Forestry, Jiamusi 154002, China

Tel: +86-454-6019151. Fax: +86-454-6019151.

mail: [cuiliy@163.cm](mailto:cuiliy@163.cm)


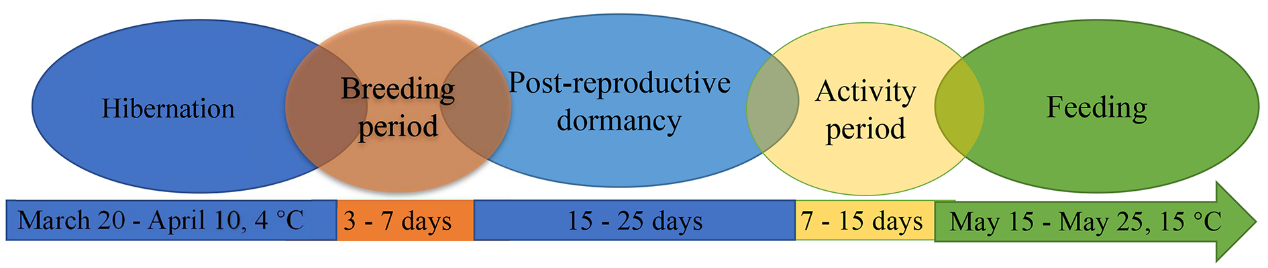


**Figure S1** Phenological time of *R. dybowskii* emerging from hibernation, post-reproduction dormancy, activity, and feeding.

After mating and spawning, both female and male *R. dybowskii* enter a dormancy period of approximately 2 weeks, and during the transitions from post-reproductive dormancy to revival and from revival to post-revival activity, *R. dybowskii* exhibits very complex behaviour that is affected by complex environmental factors.


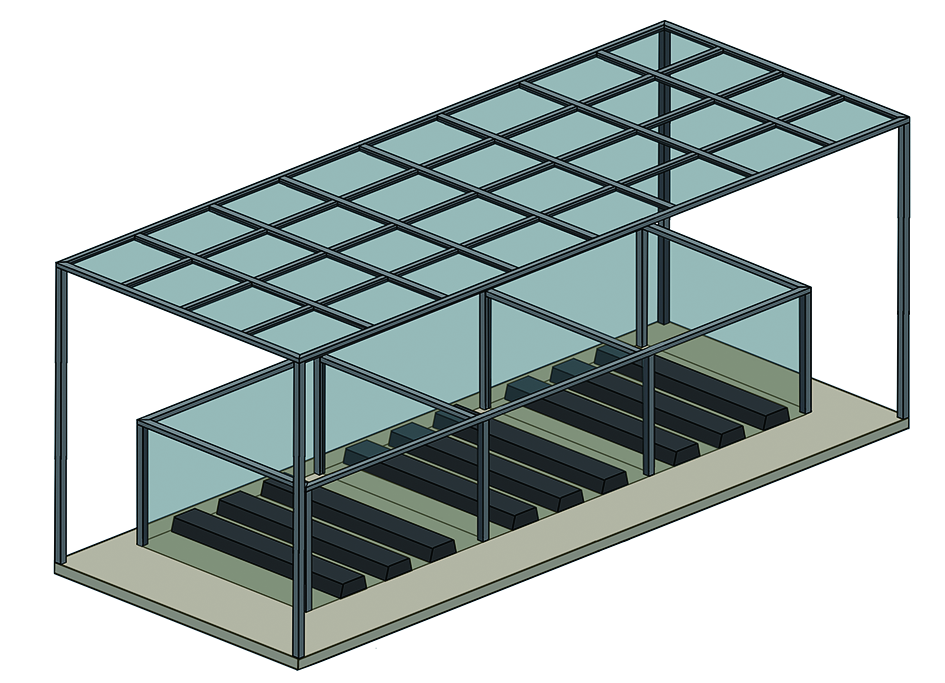


**Figure S2** Diagrams of microhabitat II described in Section 2.2.2.

Because the physical descriptions of the enclosures may be unclear, diagrams of the enclosures are provided.


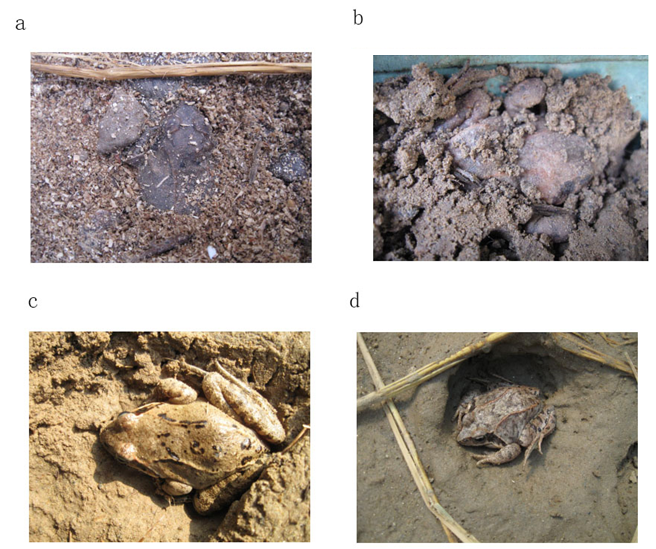


**Figure S3** The occurrence frequency of frogs was measured by their presence on the ground.

In Figure S3a, and b, the forest frog is dormant, its body curled up and its eyes tightly closed, motionless. The frogs in Figure S3b and Figure S3c, basically exposed the head and back completely, for which we estimated the exposed are was 60%. In the first few days and final few days of hibernation, most frogs were exposed as showed in Figure 34b and Figure S3c.

**
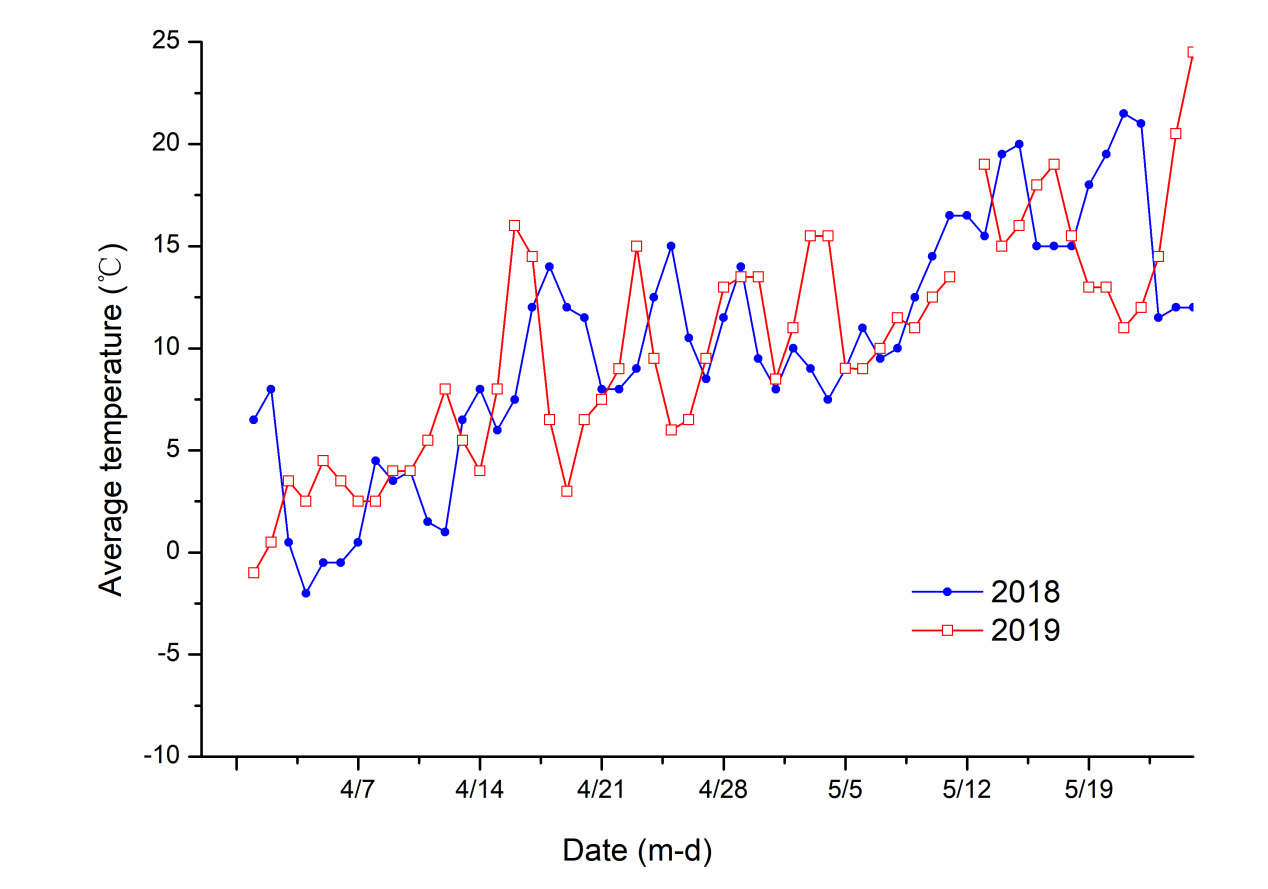
**

**Figure S4** The temperatures in the experimental enclosures.

The temperatures in the experimental enclosures all rose in a fluctuating manner from April to May.
