# Supplementary material for: Effects of fine-scale habitat quality on activity, dormancy, habitat use, and survival after reproduction in Rana dybowskii (Chordata, Amphibia)
Source: BMC Zool. 2023 Jan 16;8:1. doi: 10.1186/s40850-022-00163-4 (PMC10127375; doi:10.1186/s40850-022-00163-4)
Supplement: Supplementary file 2 — Additional file 2: Table S1. Scheirer Ray Hare test for two-factor design with shelter and shading demonstrated a statistically significant interaction for body exposure rate, body-soil contact rate, and ground hole depth. Table S2. Comparing the body exposure percentage, body-soil contact percentage and borrows depth among all 4 groups by Nemenyi test. Table S3. Used GEE to investigate the influence of shelter, shading and time by selecting the frequency of activity at four time points. Table S4. The results indicated that interaction of shelter and time, interaction of shading and time had significant. Table S5. Comparing frog behavioural differences in refuge stones and soil at four time points. Table S6. Comparisons between shade and non-shade on frog behaviour at four time points. Table S7. Used GEE to investigate the influence of shelter, shading and time by survival rate of activity at two time points. Table S8. The survival rate of frog was reduced in the post-dormancy activity period. [file 40850_2022_163_MOESM2_ESM.docx]

**Table S1** Scheirer Ray Hare test for two-factor design with shelter and shading demonstrated a statistically significant interaction for body exposure rate, body-soil contact rate, and ground hole depth.

|  | df | Sum Square | H | *P* value |
| --- | --- | --- | --- | --- |
| **body exposure rate** | | | | |
| shelter | 1 | 691012 | 145.800 | <0.001 |
| shading | 1 | 26481 | 5.600 | 0.018 |
| shelter: shading | 1 | 22485 | 4.700 | 0.029 |
| Residuals | 236 | 393065 |  |  |
|  |  |  |  |  |
| **body-soil contact rate** | | | | |
| shelter | 1 | 355740 | 73.800 | <0.001 |
| shading | 1 | 77796 | 16.100 | <0.001 |
| shelter: shading | 1 | 34728 | 7.200 | 0.007 |
| Residuals | 236 | 683679 |  |  |
|  |  |  |  |  |
| **ground hole depth** | | | | |
| shelter | 1 | 810030 | 168.100 | <0.001 |
| shading | 1 | 5472 | 1.100 | 0.287 |
| shelter: shading | 1 | 83440 | 17.300 | <0.001 |
| Residuals | 236 | 252905 |  |  |

| Comparison  Group1 VS Group2 | Group1 | | | Group2 | | | Chi-square | df | *P* value |
| --- | --- | --- | --- | --- | --- | --- | --- | --- | --- |
|  | N | Mean±SD | Median (Q1, Q3) | N | Mean±SD | Median (Q1, Q3) |  |  |  |
| **Exposure rate** | | | | | | | | | |
| Soil without shading vs Soil with shading | 60 | 7.35±7.55 | 4.50(0.00, 14.69) | 60 | 7.32±7.83 | 0.00(0.00, 15.70) | 0.017 | 3 | **0.999** |
| Soil without shading vs Stone without shading | 60 | 7.35±7.55 | 4.50(0.00, 14.69) | 60 | 23.26±7.57 | 24.10(16.10, 29.02) | 48.958 | 3 | <0.001 |
| Soil without shading vs Stone with shading | 60 | 7.35±7.55 | 4.50(0.00, 14.69) | 60 | 30.21±7.27 | 32.04(24.42, 36.00) | 104.207 | 3 | <0.001 |
| Soil with shading vs Stone without shading | 60 | 7.32±7.83 | 0.00(0.00, 15.70) | 60 | 23.26±7.57 | 24.10(16.10, 29.02) | 47.139 | 3 | <0.001 |
| Soil with shading vs Stone with shading | 60 | 7.32±7.83 | 0.00(0.00, 15.70) | 60 | 30.21±7.27 | 32.04(24.42, 36.00) | 101.544 | 3 | <0.001 |
| Stone without shading vs Stone with shading | 60 | 23.26±7.57 | 24.10(16.10, 29.02) | 60 | 30.21±7.27 | 32.04(24.42, 36.00) | 10.311 | 3 | 0.016 |
| **Body-soil contact** | | | | | | | | | |
| Soil without shading vs Soil with shading | 60 | 61.50±7.35 | 63.35(54.80, 66.26) | 60 | 61.43±4.01 | 61.15(58.68, 64.05) | 0.889 | 3 | **0.828** |
| Soil without shading vs Stone without shading | 60 | 61.50±7.35 | 63.35(54.80, 66.26) | 60 | 73.54±5.72 | 71.84(70.81, 73.62) | 63.567 | 3 | <0.001 |
| Soil without shading vs Stone with shading | 60 | 61.50±7.35 | 63.35(54.80, 66.26) | 60 | 67.26±10.61 | 67.50(59.14, 73.24) | 10.459 | 3 | 0.015 |
| Soil with shading vs Stone without shading | 60 | 61.43±4.01 | 61.15(58.68, 64.05) | 60 | 73.54±5.72 | 71.84(70.81, 73.62) | 79.489 | 3 | <0.001 |
| Soil with shading vs Stone with shading | 60 | 61.43±4.01 | 61.15(58.68, 64.05) | 60 | 67.26±10.61 | 67.50(59.14, 73.24) | 17.445 | 3 | 0.001 |
| Stone without shading vs Stone with shading | 60 | 73.54±5.72 | 71.84(70.81, 73.62) | 60 | 67.26±10.61 | 67.50(59.14, 73.24) | 22.457 | 3 | <0.001 |
| **Borrows depth** | | | | | | | | | |
| Soil without shading vs Soil with shading | 60 | 2.33±0.28 | 2.31(2.20, 2.50) | 60 | 2.09±0.35 | 2.10(1.87, 2.31) | 4.791 | 3 | **0.188** |
| Soil without shading vs Stone without shading | 60 | 2.33±0.28 | 2.31(2.20, 2.50) | 60 | 3.42±0.41 | 3.54(3.40, 3.66) | 38.751 | 3 | <0.001 |
| Soil without shading vs Stone with shading | 60 | 2.33±0.28 | 2.31(2.20, 2.50) | 60 | 3.93±0.35 | 3.95(3.72, 4.20) | 98.420 | 3 | <0.001 |
| Soil with shading vs Stone without shading | 60 | 2.09±0.35 | 2.10(1.87, 2.31) | 60 | 3.42±0.41 | 3.54(3.40, 3.66) | 70.791 | 3 | <0.001 |
| Soil with shading vs Stone with shading | 60 | 2.09±0.35 | 2.10(1.87, 2.31) | 60 | 3.93±0.35 | 3.95(3.72, 4.20) | 146.638 | 3 | <0.001 |
| Stone without shading vs Stone with shading | 60 | 3.42±0.41 | 3.54(3.40, 3.66) | 60 | 3.93±0.35 | 3.95(3.72, 4.20) | 13.658 | 3 | 0.003 |

**Table S2** Comparing the body exposure percentage, body-soil contact percentage and borrows depth among all 4 groups by Nemenyi test.

**Table S3** Used GEE to investigate the influence of shelter, shading and time by selecting the frequency of activity at four time points.

|  | Coefficient | SE | Wald | Pvalue |  |
| --- | --- | --- | --- | --- | --- |
| (Intercept) | 40.222 | 0.490 | 6735.130 | <0.001 | *** |
| Stone vs Soil | -7.556 | 0.640 | 139.440 | <0.001 | *** |
| with Shading vs without Shading | 4.556 | 0.579 | 61.890 | <0.001 | *** |
| date0425 vs date0411 | -40.056 | 0.515 | 6039.240 | <0.001 | *** |
| date0510 vs date0411 | -32.889 | 0.608 | 2924.750 | <0.001 | *** |
| date0525 vs date0411 | -15.306 | 0.590 | 672.340 | <0.001 | *** |
| Stone vs Soil:date0425 vs date0411 | 7.222 | 0.702 | 105.860 | <0.001 | *** |
| Stone vs Soil:date0510 vs date0411 | 7.222 | 0.795 | 82.580 | <0.001 | *** |
| Stone vs Soil:date0525 vs date0411 | -1.611 | 0.808 | 3.980 | 0.046 | * |
| with Shading vs without Shading:date0425 vs date0411 | -4.222 | 0.647 | 42.580 | <0.001 | *** |
| with Shading vs without Shading:date0510 vs date0411 | -9.222 | 0.747 | 152.540 | <0.001 | *** |
| with Shading vs without Shading:date0525 vs date0411 | -4.389 | 0.761 | 33.300 | <0.001 | *** |

The results indicated that interaction of shelter and time, interaction of shading and time had significant. Signif. codes: 0 ‘***’ 0.001 ‘**’ 0.01 ‘*’ 0.05 ‘.’ 0.1 ‘ ’ 1

**Table S4** The results indicated that interaction of shelter and time, interaction of shading and time had significant.

| Comparison | estimate | SE | Z | Adjusted *P*-value |
| --- | --- | --- | --- | --- |
| Soil No date0411 - Stone No date0411 | 7.556 | 0.640 | 11.809 | <0.001 |
| Soil No date0411 - Soil Yes date0411 | -4.556 | 0.579 | -7.867 | <0.001 |
| Soil No date0411 - Stone Yes date0411 | 3.000 | 1.126 | 2.665 | 0.925 |
| Soil No date0411 - Soil No date0425 | 40.056 | 0.515 | 77.713 | <0.001 |
| Soil No date0411 - Stone No date0425 | 40.389 | 0.515 | 78.359 | <0.001 |
| Soil No date0411 - Soil Yes date0425 | 39.722 | 0.643 | 61.749 | <0.001 |
| Soil No date0411 - Stone Yes date0425 | 40.056 | 0.515 | 77.713 | <0.001 |
| Soil No date0411 - Soil No date0510 | 32.889 | 0.608 | 54.081 | <0.001 |
| Soil No date0411 - Stone No date0510 | 33.222 | 0.638 | 52.083 | <0.001 |
| Soil No date0411 - Soil Yes date0510 | 37.556 | 0.694 | 54.153 | <0.001 |
| Soil No date0411 - Stone Yes date0510 | 37.889 | 0.608 | 62.303 | <0.001 |
| Soil No date0411 - Soil No date0525 | 15.306 | 0.590 | 25.930 | <0.001 |
| Soil No date0411 - Stone No date0525 | 24.472 | 0.650 | 37.650 | <0.001 |
| Soil No date0411 - Soil Yes date0525 | 15.139 | 0.755 | 20.041 | <0.001 |
| Soil No date0411 - Stone Yes date0525 | 24.306 | 0.590 | 41.177 | <0.001 |
| Stone No date0411 - Soil Yes date0411 | -12.111 | 0.471 | -25.709 | <0.001 |
| Stone No date0411 - Stone Yes date0411 | -4.556 | 0.579 | -7.867 | <0.001 |
| Stone No date0411 - Soil No date0425 | 32.500 | 0.487 | 66.800 | <0.001 |
| Stone No date0411 - Stone No date0425 | 32.833 | 0.487 | 67.485 | <0.001 |
| Stone No date0411 - Soil Yes date0425 | 32.167 | 0.620 | 51.851 | <0.001 |
| Stone No date0411 - Stone Yes date0425 | 32.500 | 0.487 | 66.800 | <0.001 |
| Stone No date0411 - Soil No date0510 | 25.333 | 0.584 | 43.390 | <0.001 |
| Stone No date0411 - Stone No date0510 | 25.667 | 0.615 | 41.752 | <0.001 |
| Stone No date0411 - Soil Yes date0510 | 3<0.001 | 0.672 | 44.623 | <0.001 |
| Stone No date0411 - Stone Yes date0510 | 30.333 | 0.584 | 51.954 | <0.001 |
| Stone No date0411 - Soil No date0525 | 7.750 | 0.565 | 13.712 | <0.001 |
| Stone No date0411 - Stone No date0525 | 16.917 | 0.627 | 26.966 | <0.001 |
| Stone No date0411 - Soil Yes date0525 | 7.583 | 0.736 | 10.304 | <0.001 |
| Stone No date0411 - Stone Yes date0525 | 16.750 | 0.565 | 29.635 | <0.001 |
| Soil Yes date0411 - Stone Yes date0411 | 7.556 | 0.640 | 11.809 | <0.001 |
| Soil Yes date0411 - Soil No date0425 | 44.611 | 0.377 | 118.297 | <0.001 |
| Soil Yes date0411 - Stone No date0425 | 44.944 | 0.377 | 119.181 | <0.001 |
| Soil Yes date0411 - Soil Yes date0425 | 44.278 | 0.539 | 82.171 | <0.001 |
| Soil Yes date0411 - Stone Yes date0425 | 44.611 | 0.377 | 118.297 | <0.001 |
| Soil Yes date0411 - Soil No date0510 | 37.444 | 0.496 | 75.437 | <0.001 |
| Soil Yes date0411 - Stone No date0510 | 37.778 | 0.532 | 70.962 | <0.001 |
| Soil Yes date0411 - Soil Yes date0510 | 42.111 | 0.598 | 70.431 | <0.001 |
| Soil Yes date0411 - Stone Yes date0510 | 42.444 | 0.496 | 85.510 | <0.001 |
| Soil Yes date0411 - Soil No date0525 | 19.861 | 0.474 | 41.874 | <0.001 |
| Soil Yes date0411 - Stone No date0525 | 29.028 | 0.547 | 53.082 | <0.001 |
| Soil Yes date0411 - Soil Yes date0525 | 19.694 | 0.669 | 29.451 | <0.001 |
| Soil Yes date0411 - Stone Yes date0525 | 28.861 | 0.474 | 60.849 | <0.001 |
| Stone Yes date0411 - Soil No date0425 | 37.056 | 0.798 | 46.463 | <0.001 |
| Stone Yes date0411 - Stone No date0425 | 37.389 | 0.798 | 46.881 | <0.001 |
| Stone Yes date0411 - Soil Yes date0425 | 36.722 | 0.886 | 41.469 | <0.001 |
| Stone Yes date0411 - Stone Yes date0425 | 37.056 | 0.798 | 46.463 | <0.001 |
| Stone Yes date0411 - Soil No date0510 | 29.889 | 0.860 | 34.740 | <0.001 |
| Stone Yes date0411 - Stone No date0510 | 30.222 | 0.882 | 34.281 | <0.001 |
| Stone Yes date0411 - Soil Yes date0510 | 34.556 | 0.923 | 37.452 | <0.001 |
| Stone Yes date0411 - Stone Yes date0510 | 34.889 | 0.860 | 40.552 | <0.001 |
| Stone Yes date0411 - Soil No date0525 | 12.306 | 0.848 | 14.514 | <0.001 |
| Stone Yes date0411 - Stone No date0525 | 21.472 | 0.890 | 24.114 | <0.001 |
| Stone Yes date0411 - Soil Yes date0525 | 12.139 | 0.970 | 12.514 | <0.001 |
| Stone Yes date0411 - Stone Yes date0525 | 21.306 | 0.848 | 25.130 | <0.001 |
| Soil No date0425 - Stone No date0425 | 0.333 | 0.289 | 1.155 | 1.000 |
| Soil No date0425 - Soil Yes date0425 | -0.333 | 0.289 | -1.155 | 1.000 |
| Soil No date0425 - Stone Yes date0425 | <0.001 | 0.136 | <0.001 | 1.000 |
| Soil No date0425 - Soil No date0510 | -7.167 | 0.394 | -18.198 | <0.001 |
| Soil No date0425 - Stone No date0510 | -6.833 | 0.438 | -15.590 | <0.001 |
| Soil No date0425 - Soil Yes date0510 | -2.500 | 0.516 | -4.845 | <0.001 |
| Soil No date0425 - Stone Yes date0510 | -2.167 | 0.394 | -5.502 | <0.001 |
| Soil No date0425 - Soil No date0525 | -24.750 | 0.366 | -67.693 | <0.001 |
| Soil No date0425 - Stone No date0525 | -15.583 | 0.456 | -34.189 | <0.001 |
| Soil No date0425 - Soil Yes date0525 | -24.917 | 0.597 | -41.766 | <0.001 |
| Soil No date0425 - Stone Yes date0525 | -15.750 | 0.366 | -43.077 | <0.001 |
| Stone No date0425 - Soil Yes date0425 | -0.667 | 0.561 | -1.188 | 1.000 |
| Stone No date0425 - Stone Yes date0425 | -0.333 | 0.289 | -1.155 | 1.000 |
| Stone No date0425 - Soil No date0510 | -7.500 | 0.394 | -19.044 | <0.001 |
| Stone No date0425 - Stone No date0510 | -7.167 | 0.438 | -16.350 | <0.001 |
| Stone No date0425 - Soil Yes date0510 | -2.833 | 0.516 | -5.491 | <0.001 |
| Stone No date0425 - Stone Yes date0510 | -2.500 | 0.394 | -6.348 | <0.001 |
| Stone No date0425 - Soil No date0525 | -25.083 | 0.366 | -68.604 | <0.001 |
| Stone No date0425 - Stone No date0525 | -15.917 | 0.456 | -34.920 | <0.001 |
| Stone No date0425 - Soil Yes date0525 | -25.250 | 0.597 | -42.325 | <0.001 |
| Stone No date0425 - Stone Yes date0525 | -16.083 | 0.366 | -43.989 | <0.001 |
| Soil Yes date0425 - Stone Yes date0425 | 0.333 | 0.289 | 1.155 | 1.000 |
| Soil Yes date0425 - Soil No date0510 | -6.833 | 0.551 | -12.409 | <0.001 |
| Soil Yes date0425 - Stone No date0510 | -6.500 | 0.583 | -11.143 | <0.001 |
| Soil Yes date0425 - Soil Yes date0510 | -2.167 | 0.644 | -3.366 | 0.092 |
| Soil Yes date0425 - Stone Yes date0510 | -1.833 | 0.551 | -3.329 | 0.104 |
| Soil Yes date0425 - Soil No date0525 | -24.417 | 0.531 | -45.993 | <0.001 |
| Soil Yes date0425 - Stone No date0525 | -15.250 | 0.597 | -25.563 | <0.001 |
| Soil Yes date0425 - Soil Yes date0525 | -24.583 | 0.710 | -34.626 | <0.001 |
| Soil Yes date0425 - Stone Yes date0525 | -15.417 | 0.531 | -29.040 | <0.001 |
| Stone Yes date0425 - Soil No date0510 | -7.167 | 0.394 | -18.198 | <0.001 |
| Stone Yes date0425 - Stone No date0510 | -6.833 | 0.438 | -15.590 | <0.001 |
| Stone Yes date0425 - Soil Yes date0510 | -2.500 | 0.516 | -4.845 | <0.001 |
| Stone Yes date0425 - Stone Yes date0510 | -2.167 | 0.394 | -5.502 | <0.001 |
| Stone Yes date0425 - Soil No date0525 | -24.750 | 0.366 | -67.693 | <0.001 |
| Stone Yes date0425 - Stone No date0525 | -15.583 | 0.456 | -34.189 | <0.001 |
| Stone Yes date0425 - Soil Yes date0525 | -24.917 | 0.597 | -41.766 | <0.001 |
| Stone Yes date0425 - Stone Yes date0525 | -15.750 | 0.366 | -43.077 | <0.001 |
| Soil No date0510 - Stone No date0510 | 0.333 | 0.471 | 0.707 | 1.000 |
| Soil No date0510 - Soil Yes date0510 | 4.667 | 0.471 | 9.899 | <0.001 |
| Soil No date0510 - Stone Yes date0510 | 5.000 | 0.544 | 9.186 | <0.001 |
| Soil No date0510 - Soil No date0525 | -17.583 | 0.488 | -36.054 | <0.001 |
| Soil No date0510 - Stone No date0525 | -8.417 | 0.558 | -15.070 | <0.001 |
| Soil No date0510 - Soil Yes date0525 | -17.750 | 0.678 | -26.169 | <0.001 |
| Soil No date0510 - Stone Yes date0525 | -8.583 | 0.488 | -17.600 | <0.001 |
| Stone No date0510 - Soil Yes date0510 | 4.333 | 0.770 | 5.629 | <0.001 |
| Stone No date0510 - Stone Yes date0510 | 4.667 | 0.471 | 9.899 | <0.001 |
| Stone No date0510 - Soil No date0525 | -17.917 | 0.524 | -34.173 | <0.001 |
| Stone No date0510 - Stone No date0525 | -8.750 | 0.591 | -14.812 | <0.001 |
| Stone No date0510 - Soil Yes date0525 | -18.083 | 0.705 | -25.648 | <0.001 |
| Stone No date0510 - Stone Yes date0525 | -8.917 | 0.524 | -17.007 | <0.001 |
| Soil Yes date0510 - Stone Yes date0510 | 0.333 | 0.471 | 0.707 | 1.000 |
| Soil Yes date0510 - Soil No date0525 | -22.250 | 0.591 | -37.665 | <0.001 |
| Soil Yes date0510 - Stone No date0525 | -13.083 | 0.650 | -20.116 | <0.001 |
| Soil Yes date0510 - Soil Yes date0525 | -22.417 | 0.756 | -29.661 | <0.001 |
| Soil Yes date0510 - Stone Yes date0525 | -13.250 | 0.591 | -22.430 | <0.001 |
| Stone Yes date0510 - Soil No date0525 | -22.583 | 0.488 | -46.306 | <0.001 |
| Stone Yes date0510 - Stone No date0525 | -13.417 | 0.558 | -24.023 | <0.001 |
| Stone Yes date0510 - Soil Yes date0525 | -22.750 | 0.678 | -33.541 | <0.001 |
| Stone Yes date0510 - Stone Yes date0525 | -13.583 | 0.488 | -27.852 | <0.001 |
| Soil No date0525 - Stone No date0525 | 9.167 | 0.493 | 18.593 | <0.001 |
| Soil No date0525 - Soil Yes date0525 | -0.167 | 0.493 | -0.338 | 1.000 |
| Soil No date0525 - Stone Yes date0525 | 9.000 | 0.436 | 20.657 | <0.001 |
| Stone No date0525 - Soil Yes date0525 | -9.333 | 0.885 | -10.552 | <0.001 |
| Stone No date0525 - Stone Yes date0525 | -0.167 | 0.493 | -0.338 | 1.000 |
| Soil Yes date0525 - Stone Yes date0525 | 9.167 | 0.493 | 18.593 | <0.001 |

**Table S5** Comparing frog behavioural differences in refuge stones and soil at four time points.

| Contrast | Coefficient | SE | Z | *Adjust P*value |
| --- | --- | --- | --- | --- |
| Soil without shading date0411 - Stone without shading date0411 | 7.556 | 0.640 | 11.809 | 0.000 |
| Soil with shading date0411 - Stone with shading date0411 | 7.556 | 0.640 | 11.809 | 0.000 |
| Soil without shading date0425 - Stone without shading date0425 | 0.333 | 0.289 | 1.155 | 1.000 |
| Soil with shading date0425 - Stone with shading date0425 | 0.333 | 0.289 | 1.155 | 1.000 |
| Soil without shading date0510 - Stone without shading date0510 | 0.333 | 0.471 | 0.707 | 1.000 |
| Soil without shading date0510 - Stone without shading date0525 | -8.417 | 0.558 | -15.070 | 0.000 |
| Soil with shading date0510 - Stone with shading date0510 | 0.333 | 0.471 | 0.707 | 1.000 |
| Soil with shading date0525 - Stone with shading date0525 | 9.167 | 0.493 | 18.593 | 0.000 |

**Table S6** Comparisons between shade and non-shade on frog behaviour at four time points.

| Contrast | Estimate | SE | Z | *Adjust P*value |
| --- | --- | --- | --- | --- |
| Soil without shading date0411 - Soil with shading date0411 | -4.556 | 0.579 | -7.867 | 0.000 |
| Stone without shading date0411 - Stone with shading date0411 | -4.55556 | 0.579 | -7.86711 | 4.36E-13 |
| Soil without shading date0425 - Soil with shading date0425 | -0.333 | 0.289 | -1.155 | 1.000 |
| Stone without shading date0425 - Stone with shading date0425 | -0.333 | 0.289 | -1.155 | 1.000 |
| Soil without shading date0510 - Soil with shading date0510 | -17.750 | 0.678 | -26.169 | 0.000 |
| Stone without shading date0510 - Stone with shading date0510 | -0.167 | 0.493 | -0.338 | 1.000 |
| Stone without shading date0525 - Stone with shading date0525 | -6.83333 | 0.550 | -12.4091 | 2.8E-33 |
| Soil without shading date0525 - Soil with shading date0525 | -13.5833 | 0.487 | -27.8521 | 1.2E-168 |

**Table S7** Used GEE to investigate the influence of shelter, shading and time by survival rate of activity at two time points.

|  | Coefficient | SE | Wald | Pvalue |  |
| --- | --- | --- | --- | --- | --- |
| (Intercept) | 81.170 | 3.220 | 634.710 | <0.001 | *** |
| Stone vs Soil | 4.670 | 2.410 | 3.760 | 0.052 | . |
| with shading vs without shading | -1.670 | 2.410 | 0.480 | 0.489 |  |
| date0525 vs date0506 | -19.330 | 2.410 | 64.540 | <0.001 | *** |

Signif. codes: 0 ‘***’ 0.001 ‘**’ 0.01 ‘*’ 0.05 ‘.’ 0.1 ‘ ’ 1

**Table S8** The survival rate of frog was reduced in the post-dormancy activity period.

| Contrast | Estimate | SE | Z | *Adjust P*value |
| --- | --- | --- | --- | --- |
| date0506 vs date0525 | 19.333 | 2.407 | 8.034 | 0.000 |
